# Managing of Dyslipidaemia Characterized by Accumulation of Triglyceride-Rich Lipoproteins

**DOI:** 10.1007/s11883-022-00979-y

**Published:** 2022-02-02

**Authors:** Jolien Visser, Willemien van Zwol, Jan Albert Kuivenhoven

**Affiliations:** Department of Pediatrics, University Medical Centre Groningen, University of Groningen, Groningen, The Netherlands

**Keywords:** Atherosclerotic cardiovascular disease, Pancreatitis, Remnant cholesterol, Hypertriglyceridemia, TRL, Triglyceride-lowering drugs

## Abstract

**Purpose of Review:**

The accumulation of triglyceride-rich lipoproteins (TRLs) in plasma in patients with familial chylomicronaemia syndrome (FCS) or severe hypertriglyceridemia is associated with an increased risk of potentially life-threatening pancreatitis. Elevated TRL levels have also been suggested to contribute to atherosclerotic cardiovascular disease (ASCVD). This review provides the latest progress that has been made in this field of research.

**Recent Findings:**

Apolipoprotein C-III and angiopoietin-like protein 3 play key roles in the metabolism of TRLs. Targeting their production in the liver or their presence in the circulation effectively reduces triglycerides in patients with FCS or severe hypertriglyceridemia. Attempts to reduce triglyceride synthesis in the small intestine have been halted. Early studies with a fibroblast growth factor 21 agonist have shown to reduce plasma triglycerides and hepatic steatosis and improve glucose homeostasis.

**Summary:**

New drugs have recently been shown to effectively reduce plasma triglycerides which render hope for treating the risk of pancreatitis. Studies that have just been initiated will learn whether this unmet clinical will be met. It is too early to evaluate the potential of these drugs to reduce the risk of atherosclerosis through the reduction of triglycerides.

## Introduction

Hypertriglyceridemia, characterized by elevated levels of plasma triglycerides, is a common and growing health care burden [1]. The current strong interest in strategies to lower triglycerides in plasma is largely driven by the concept that this may attenuate atherosclerosis on top of cholesterol-lowering drugs. The support for this idea is provided by genetic studies that have recently suggested a causal link between increased levels of triglycerides and the risk of atherosclerotic cardiovascular disease (ASCVD) [2–6].

The metabolism of triglycerides is complex but detailed in several excellent recent reviews [1, 6]. Here, we only shortly describe the key pathways of triglyceride metabolism. Dietary triglycerides are taken up by the small intestine where they are repackaged in large macromolecules known as chylomicrons. With triglycerides as their main component, chylomicrons are secreted into the lymph from where they reach the systemic circulation via the superior vena cava in the heart. Depending on the metabolic demand, the liver secretes smaller triglyceride-containing macromolecules known as very low-density lipoproteins (VLDL). In the periphery, the triglycerides packed in chylomicrons as well as in VLDL are lipolyzed and taken up for energy production (skeletal muscle) or storage (fat tissue). The remaining smaller chylomicron remnants and VLDL remnants are cleared by the liver. VLDL and chylomicrons are commonly referred to as triglyceride-rich lipoproteins (TRLs) [6].

TRLs have been proposed to increase the risk of ASCVD via, e.g. endothelial dysfunction in peripheral and coronary arteries [7]. Following initial lipolysis, remnant TRLs have also been reported to pass the endothelium and reach the intima where they can contribute to the formation of lipid laden monocyte-macrophages, the culprit of atherosclerosis [8]. In addition, cholesterol in TRLs remnants has also been associated with low-grade inflammation [9].

Current drugs that lower low-density lipoprotein (LDL) cholesterol only have moderate effects on triglyceride levels [1]. While fibrates reduce plasma triglycerides, there is no definitive proof that they lower the risk of atherosclerosis. Fortunately, there is positive news on the use of fish oils and derivate which will be discussed elsewhere in this series. The current review will primarily focus on drugs that lower plasma triglyceride levels via targeting the hepatic production of lipoproteins and/or increasing their catabolism.

The development of triglyceride-lowering drugs that reduce atherosclerosis is welcome when considering the increasing number of metabolic patients suffering from obesity, non-alcoholic fatty liver disease (NAFLD) and type 2 diabetes mellitus (T2DM) who are at increased risk of ASCVD and mostly present with mild hypertriglyceridemia [10]. The drugs that are currently being used to treat hypertriglyceridemia are, however, not tested in patients at increased risk for ASCVD but mostly in patients with familial chylomicronaemia syndrome (FCS) or severe hypertriglyceridemia. As already indicated, this is a condition that is associated with an increased risk of pancreatitis [11]. This clinical complication is commonly seen in rare autosomal recessive Mendelian disorders of lipid metabolism (*LPL*, *APOC2*, *APOA5*, *GPIHBP1*, *LMF1, GPD1*) [11]. Loss-of-function mutations in one or more of these genes can cause a complete loss of lipolysis of triglycerides in TRLs. Patients suffering from these genetic defects are diagnosed with FCS. In addition to these monogenic disorders, numerous smaller effect size variants in multiple genes known as polygenic hypertriglyceridemia can also lead to severe hypertriglyceridemia [12]. While it has long been accepted that the risk of pancreatitis was becoming real at triglycerides above 888 mg/dL (10 mmol/l), more recent data show that a non-fasting triglycerides of > 177 mg are already associated with higher risk of acute pancreatitis [13]. This finding illustrates that the management of (severe) hypertriglyceridemia may benefit more individuals than previously anticipated.

Effective treatment of severe hypertriglyceridemia is a long-standing unmet need as sufficient lifestyle adjustment (less than 10% of fat of total caloric intake) is very challenging if not impossible to comply with by patients suffering from FCS [14]. Thus far, only one drug was registered for the treatment of severe hypertriglyceridemia which concerns gene therapy for patients suffering from lipoprotein lipase (LPL) deficiency [15]. However, 2 years after EMA approval, it was withdrawn from the market in 2017. Fortunately, over the last few years multiple promising strategies to lower triglycerides have been developed which are at the centre of this review.

## Lowering Triglycerides Through Targeting the Anabolism and/or Catabolism of Lipoproteins

This section provides details on the efforts to lower plasma triglyceride levels via targeting the hepatic production of lipoproteins and/or increasing their catabolism. Results of these studies are summarized in Table 1.

**Table 1:** Overview of triglyceride-lowering drugs in clinical trials

Target	Drug & mode of action	Phase of development	Targeted populations	Average reduction in plasma TG levels from baseline	References
APOC3	Volanesorsen *ASO to block ApoC-III synthesis* Conditionally approved by EMA for FCS in 2019	Phase 3 (NCT02211209)	FCS (N = 66; fasting TG ≥ 750 mg/dL)	-77% (after 13 weeks)	Witztum et al. (2019) [20]
		Phase 3 (NCT02300233)	Severe hypertriglyceridemia (N = 86; fasting TG ≥ 500 mg/dL)	-71% (after 13 weeks)	Gouni-Berthold et al. (2021) [19]
	AKCEA-APOCIII-LRx* *ASO to block ApoC-III synthesis*	Phase 3 (NCT04568434)	FCS (N = 60)	Completed in June 2023	NCT04568434
		Phase 2 (NCT03385239)	Hypertriglyceridemia and established CVD (N = 114; fasting TG 200–500 mg/dL)	-62% [50 mg] (after 6 months)	Ionis pharmaceuticals (2020) [23•]
		Phase 1/2a (NCT02900027)	Healthy volunteers with elevated TGs (N = 16; fasting TG ≥ 200 mg/dL)	-73% to -77% [90–120 mg] (after 14 days)	Alexander et al. (2019) [22]
	ARO-APOC3* *siRNAs to block ApoC-III synthesis*	Phase 3	FCS	Expected to start in 2021	A) Arrowhead pharmaceuticals (2021) [26]
		Phase 2b	Mixed dyslipidaemia	Expected to start in 2021	Arrowhead pharmaceuticals (2021) [26]
		Phase 2b (NCT04720534)	Severe hypertriglyceridemia (N = 300; fasting TG ≥ 500 mg/dL)	Completed in July 2022	Arrowhead pharmaceuticals (2021) [26] NCT04720534
		Phase 1/2a (NCT03783377)	Healthy volunteers (n = 40; fasting TG ≥ 80 mg/dL) and Severe hypertriglyceridemia (n = 3; fasting TG ≥ 300 mg/dL)**	Healthy volunteers: -53% to -64% [10–100 mg] (after 4 weeks); Severe hypertriglyceridaemic patients: -95% [50 mg] (after 29 days)	Ballantyne (2020) [25], Arrowhead pharmaceuticals (2020) [27••]
	STT-5058 *Monoclonal antibody targets ApoC-III in plasma*	Phase 1 (NCT04419688)	Healthy volunteers (N = 104; fasting TG ≥ 70 mg/dL to ≤ 400 mg/dL)	Completed in May 2021	NCT04419688
ANGPTL3	Evinacumab *Monoclonal antibody targets ANGPTL3 in plasma* FDA approved for HoFH in 2021	Phase 3 (NCT03399786)	HoFH (N = 65; Median baseline TGs = 91 mg/dL)	-55% (after 24 weeks)	Raal et al. (2020) [34•]
		Phase 2 (NCT04863014)	Severe Hypertriglyceridemia (n = 120; fasting TG > 880 mg/dL)	Expected to start in 2021	NCT04863014
		Phase 2 (NCT03452228)	Severe Hypertriglyceridemia (n = 51; fasting TG at screening ≥ 500 mg/dL and history of fasting TG ≥ 1000 mg/dL of more than 1 occasion	-57% (after 12 weeks)	Rosenson et al. (2021) [35••]
		Phase 2 (NCT02265952)	HoFH (N = 9)	-47% (after 4 weeks)	Gaudet et al. (2017) [33]
	IONIS-ANGPTL3-LRx* *ASO to block ANGPTL3 synthesis*	Phase 2b (NCT04516291)	Dyslipidaemia, Hyperlipidaemia, Hyperlipoproteinemia (N = 260; fasting TG ≥ 150 to ≤ 500 mg/dL; fasting non-HDL-C ≥ 100 mg/dL)	Completed in 2022	NCT04516291
		Phase 2 (NCT03371355)	Hypertriglyceridemia, T2DM and NAFLD (N = 105; fasting TG ≥ 150 mg/dL)	-36% to -53% [20–80 mg] (after 27 weeks)	Gaudet et al. (2020) [37•],
		Phase 1 (NCT02709850)	Healthy volunteers (N = 44; fasting TG 90–150 or ≥ 150 mg/dL)	-33% to -63% [10–60 mg] (after 6 weeks)	Graham et al. (2017) [36]
	ARO-ANG3* *SiRNAs to block ANGPTL3 synthesis*	Phase 2b (NCT04832971)	Dyslipidaemia, FCS, Hypertriglyceri-demia (N = 180; fasting TG ≥ 150 mg/dL to ≤ 500 mg/dL; LDL-C ≥ 70 mg/dL or non-HDL-C ≥ 100 mg/dL)	Completed in May 2021	NCT04832971
		Phase 1 (NCT03747224)	Healthy volunteers (n = 40) and Severe hypertriglyceridemia (n = 5)**	Healthy volunteers: -47% to -53% [35–200 mg] (after 8 weeks) Severe hypertriglyceridemic patients: -79% [200 mg] (after 29 days)	Watts (2020) [38], Arrowhead pharmaceuticals (2020) [27••]
DGAT1	Pradigastat *DGAT1 inhibitor*	Phase 3 (NCT01589237)	FCS (N = 38)	Prematurely terminated in 2015***	NCT01589237
		Phase 2 (NCT04620161)	Functional constipation (N = 180)	Completed in May 2022	NCT04620161
		Phase 2 (NCT01474434)	CAD and hypertriglyceridemia (N = 41)	Prematurely terminated in 2014***	NCT01474434
		Phase 2 (NCT01146522)	FCS (N = 8)	-41% to -70% [20–40 mg] (after 3 weeks)	Meyers et al. (2015) [46]
FGF21	BIO89-100 *GlycoPEGylated analog of FGF21*	Phase 2b/3	Fibrosis stage 2 or 3 NASH	Expected to start in 2021	89BIO (2021) [50]
		Phase 2 (NCT04541186)	Severe hypertriglyceridemia (N = 90; fasting TG ≥ 500 mg/dL and ≤ 2000 mg/dL)	Completed in November 2021	NCT04541186
		Phase 1b/2a (NCT04048135)	NAFLD patients at high risk of NASH or and NASH patients (N = 71)	-18% to -28% [3–36 mg] In subgroup with baseline TG ≥ 200 mg/dL) (-33% to -49% [3–36 mg] (after 13 weeks)	Frias et al. (2021) [49••]
		Phase 1a	Healthy volunteers (N = 46)	-33 to -51% [9–78 mg] (after 8 days)	89BIO (2020) [48]

Abbreviations: APOC3, apolipoprotein C-III; ANGPTL3, angiopoietin-like protein 3; ASO, antisense oligonucleotide; CVD, cardiovascular disease; DGAT1, diacylglycerol acyltransferase 1; EMA, European medicines agency; FCS, Familial Chylomicronemia Syndrome; FDA, food and drug administration; FGF21, fibroblast growth-factor 21; GalNAc, N-acetylgalactosamine; HoFH, Homozygous Familial Hypercholesterolemia; NAFLD, Non-alcoholic Fatty Liver Disease; NASH, Non-alcoholic Steatohepatitis; siRNA, single-interfering RNA; TG, triglyceride

^*^GalNAc modification: GalNAc (N-acetylgalactosamine) binds to the Asialoglycoprotein receptor that is highly expressed on hepatocytes, enhancing hepatic uptake of the drug

^**^ Trial NCT03747224 The number of patients in each patient group and triglyceride inclusion criteria were not described

^***^Pradigastat—The clinical trials pages do not provide information on the actual reason for termination

### APOC3 Antagonists

*APOC3* encodes for apolipoprotein (apo)C-III in the small intestine and liver from where it is secreted with TRLs. Bound to the surface of TRL, it is thought to inhibit lipoprotein lipase-mediated triglyceride lipolysis and to interfere with the hepatic clearance of TRL [16]. Genetic studies have been a cornerstone to the development of *APOC3* antagonists: carriers of loss-of-function mutations in *APOC3* have 39% lower plasma triglyceride levels and a 40% reduction in ASCVD risk compared to non-carriers [17].

**Fig. 1:**
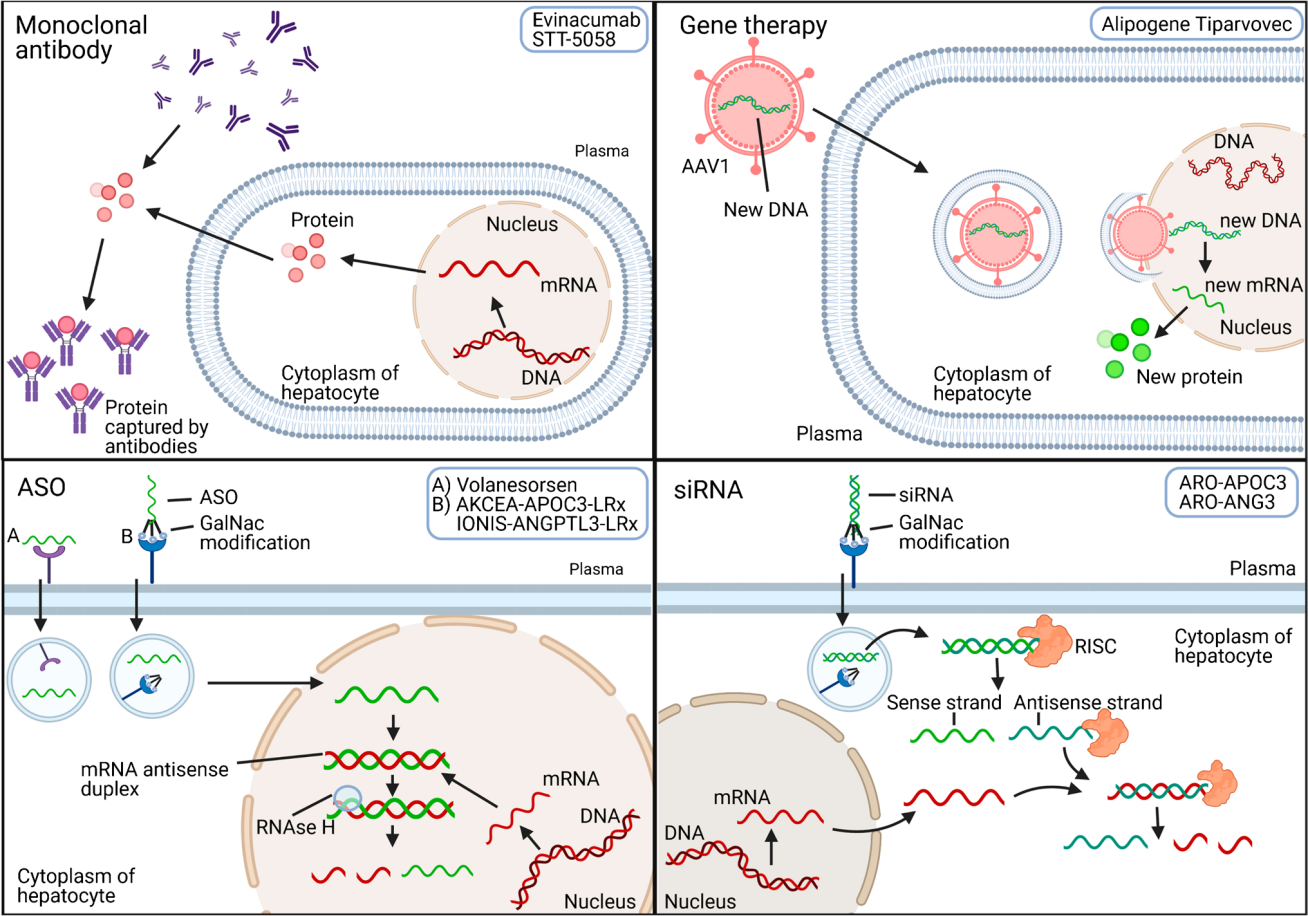
Therapies to lower triglycerides in plasma with the use of monoclonal antibodies, gene therapy, antisense oligonucleotides (ASO) and single-interfering RNA (siRNA). Monoclonal Antibody: Monoclonal antibodies are injected into the circulation where they bind and thereby inactivate their target proteins. Gene therapy: For LPL gene therapy, adeno-associated virus (AAV) subtype 1 was used as a vector to transduce skeletal muscle cells to produce LPL. The virus releases its cargo, i.e. the LPL cDNA into the nucleus, where mRNA can be transcribed and translated into LPL protein. Antisense oligonucleotide (ASO): A single-strand ASO (with or without GalNAc* modification) is generally injected subcutaneously and is taken up via endocytosis from the circulation. In the cytoplasm or after entering the nucleus, the antisense oligonucleotide binds to the complementary sequence of the targeted mRNA. RNase H recognizes the resulting mRNA antisense duplex and cleaves the mRNA and prevents protein translation of the targeted protein. Single interfering RNA (siRNA): A double-stranded siRNA with GalNac* modification is injected subcutaneous and taken up via endocytosis. In the cytoplasm, it is recognized by the RNA-induced silencing complex (RISC) which removes the sense strand. The resulting complex binds to the complementary mRNA sequence in the cytoplasm and degrades it, thereby preventing target protein translation. *GalNAc modification: increases specificity for uptake in liver cells by binding the asialoglycoprotein receptor (ASGR). This image has been generated in BioRender

Volanesorsen was the first *APOC3* antagonist to achieve FDA approval in 2019 and has thus far only been used for the treatment of FCS. It concerns an antisense oligonucleotide (ASO), a single-stranded deoxyribonucleotide that is complementary to the target mRNA. After binding, it prompts the degradation of the *APOC3* mRNA, thereby blocking translation into protein (Fig. 1) [18]. ASOs are generally administered through subcutaneous injections. Phase 3 clinical trials have shown that Volanesorsen can reduce triglyceride levels with 71% and 77% in patients with severe hypertriglyceridemia (fasting triglycerides ≥ 500 mg/dL) and FCS (fasting triglycerides ≥ 750 mg/dL), respectively [19, 20]. In the APPROACH trial, in which 66 patients with FCS were tested, thrombocytopenia was a common adverse event [20]. This unexpected result raised the question whether thrombocytopenia is an on-target effect caused by reduced apoC-III production or an off-target effect of the respective ASO. An answer to this question was provided after testing of a N-acetylgalactosamine (GalNAc) conjugated ASO (AKCEA-APOC3-LRx). This modification renders a more specific delivery of the ASO to the liver: GalNAc binds to the asialoglycoprotein receptor that is highly expressed on hepatocytes leading to efficient hepatic uptake [21]. Following a successful dose titration study in healthy volunteers [22], AKCEA-APOC3-LRx was shown to reduce triglycerides by 62% with a monthly 50 mg subcutaneous injection in a Phase 2 trial with 114 patients with mild hypertriglyceridemia [23•] (baseline triglyceride levels were not provided). Notably, 91% of the patients achieved triglyceride levels of < 150 mg/dL at this dose at 6 months [23•]. No patients developed thrombocytopenia during this 52-week study and thus, the previously noted thrombocytopenia was most likely an off-target effect of Volanesorsen and not caused by a loss of hepatic apoC-III secretion and/or its downstream effects. It is likely that Volanesorsen will be replaced by AKCEA-APOC3-LRx in the near future. To date, patients who are treated with Volanesorsen are closely monitored by a physician, receive platelet counts at least every 2 weeks and treatment is discontinued when platelet levels are too low [24]. Currently, a phase 3 trial with AKCEA-APOC3-LRx in patients with FCS is ongoing of which results are expected in 2023.

In addition, a siRNA-based strategy has been developed to reduce hepatic apoC-III production (Fig. 1). A double-stranded siRNA is recognized by the RNA-induced silencing complex in the cytoplasm that removes the sense strand. The resulting complex binds to the complementary mRNA sequence in the cytoplasm and degrades it, thereby preventing target protein translation [18]. A phase 1/2a single and multiple-dose 16-week study was conducted to evaluate the safety, tolerability, pharmacokinetics and pharmacodynamic effects of ARO-APOC3 in 40 healthy volunteers (fasting triglycerides ≥ 80 mg/dL) and 3 subjects with fasting triglycerides ≥ 300 mg/dL. Preliminary results showed that triglycerides were reduced up to 64% using a single subcutaneous injection [100 mg] and up to 92% after two dosages [25, 26]. In addition, ARO-APOC3 reduced triglycerides levels by 95% [50 mg] in the severe hypertriglyceridaemic patients [27••]. A phase 2b study in 300 severe hypertriglyceridaemic patients (fasting triglycerides ≥ 500 mg/dL) is ongoing and additional studies are planned to start in 2021 which include a phase 2b dose-finding study in patients with mixed dyslipidaemia and a phase 3 clinical study in patients with FCS [26].

The most recently developed *APOC3* antagonist is STT-5058, a monoclonal antibody that targets apoC-III in the circulation (Fig. 1). This drug is currently tested in a Phase 1 clinical trial in 104 healthy volunteers with fasting triglycerides ≥ 70 to ≤ 400 mg/dL (NCT04419688). The treatment consists of 3 doses of intravenous injections at 2-week intervals, up to 6 doses in total. The study is expected to be completed in Q2 (refers to May 2021).

Taken together, APOC3 antagonists have so far been tested in patients with FCS and severe hypertriglyceridemia with pronounced reductions in plasma triglycerides.

### ANGPTL3 Antagonists

Although angiopoietin-like protein 3, ANGPTL3, was shown to play a major role in lipid metabolism in KK/San mice in 2002 [28], the discovery that human genetic *ANGPTL3* deficiency is associated with reductions in all plasma lipids sparked a large pharmaceutical interest in this protein [29]. This interest was initially focused on the potency of ANGPTL3 antagonists to reduce LDL cholesterol but more recently also triglycerides. Secreted by the liver into the circulation, ANGPTL3 is mostly known as an inhibitor of both LPL and endothelial lipase which play key roles in the lipolysis of lipoprotein-associated triglycerides and phospholipids, respectively [30]. New evidence suggests that ANGPTL3 affects a novel endothelial lipase-dependent pathway that lowers LDL cholesterol through endothelial lipase-dependent VLDL clearance, but how ANGPTL3 exactly affects lipid and lipoprotein metabolism remains elusive [31].

In February 2021, Evinacumab, a monoclonal antibody against ANGPTL3 (Fig. 1) was approved by the FDA for the treatment of homozygous familial hypercholesterolemia (HoFH) as an adjunct to existing LDL cholesterol-lowering therapies. ANGPTL3 inactivation lowers LDL cholesterol, in contrast to statins and PCSK9 antagonists, independently of the classical LDL receptor-mediated pathway and thus represents a promising therapeutic approach for individuals with HoFH characterized by a complete absence of LDLR activity [32]: In HoFH patients, the drug at 15 mg/kg bodyweight has been shown to markedly reduce LDL cholesterol by 49% with simultaneous decreases in triglyceride levels up to 55% compared to baseline [34•].

New data presented at American College of Cardiology meeting (ACC 2021) revealed that Evinacumab holds promise for patients with severe hypertriglyceridemia [35••]. A phase 2 trial enrolled 51 patients that had triglyceride levels of > 500 mg/dL and a history of prior hospitalization for acute pancreatitis. All participants underwent genetic testing to assess mutations in genes involved in LPL-mediated triglyceride hydrolysis. After 12 weeks, the median triglyceride levels dropped by more than 800 mg/dL (57%) in patients taking Evinacumab. Interestingly, the magnitude of triglyceride-lowering was dependent on the participants’ genetic profile. Those suffering from multiple mutations in genes involved in LPL-mediated triglyceride hydrolysis had essentially no benefit from Evinacumab, whereas patients with no, or a single mutation in respective genes, were seen to have triglyceride reductions around 80% [35••].

Another new phase 2 clinical study to evaluate the efficacy and safety of Evinacumab in 120 patients with severe hypertriglyceridemia (fasting triglycerides > 880 mg/dL) for the prevention of recurrent acute pancreatitis is planned to start in July 2021 (NCT04863014). These findings and announcements give hope that Evinacumab could also be made available for other patient groups besides HoFH in the (near) future.

In parallel to Evinacumab, a GalNAc-conjugated ASO against ANGPTL3, IONIS-ANGPTL3_LRx, has been developed. Results from a phase 1 trial have shown up to 63% lower triglyceride levels in healthy volunteers following weekly subcutaneous injections [60 mg] [36]. Data from a 27-week phase 2 trial in 105 subjects with hypertriglyceridemia, T2DM and NAFLD who received monthly or weekly injections, showed that IONIS-ANGPTL3_LRx decreased triglycerides with 36 to 53% [20–80 mg] [37•]. Unlike the studies discussed so far, this study targets cardiometabolic patients and not patients with severe dyslipidaemia. However, no reductions in liver fat or decreases in haemoglobin A1c (HbA1c) were observed [37•]. Currently, participants are being recruited for a 2b dose-ranging study in 260 patients with dyslipidaemia, hyperlipidaemia or hyperlipoproteinemia (fasting triglycerides ≥ 150 to ≤ 500 mg/dL; fasting non-HDL cholesterol ≥ 100 mg/dL) who are receiving statins. This study will address the question whether this drug may be an attractive option for cardiometabolic patients who are at increased risk of ASCVD.

ARO-ANG3, an RNAi drug, is currently the third means used to target ANGPTL3 (Fig. 1). The drug silences ANGPTL3 mRNA in the liver and has been shown to decrease triglycerides by 47% [35 mg] to 53% [200 mg] after a single subcutaneous injection in 40 normolipidemic volunteers [38]. ARO-ANG3 has also been shown to lower LDL cholesterol by 39–42% [100–300 mg] in 22 hypercholesteraemic patients on statins, with or without ezetimibe, and some receiving PCSK9 inhibitors [27••]. In addition, 5 patients with severe hypertriglyceridemia treated ARO-ANG3 reduced triglycerides up to 79% [200 mg] [27••].

These studies combined show that ANGPTL3 antagonists have the potential to markedly reduce LDL cholesterol and triglycerides in various clinical settings except for patients suffering from multiple mutations in genes encoding for proteins involved in LPL-mediated triglyceride hydrolysis. Early results do not show additional positive effects on glucose metabolism and NAFLD.

## Alternative Options to Lowering Triglycerides

The use of fibrates, fish oils, synthetic derivate and Lomitapide will be described elsewhere in this issue. Although the latter drug is used in a clinical setting to treat HoFH, it is also has been shown to reduce the risk of pancreatitis in a patient suffering from LPL deficiency [39]. Below, we describe three additional approaches that have been or are specifically tested to treat severe hypertriglyceridemia.

### Gene Therapy

Alipopogene Tiparvovec (Glybera), a gene therapy approach for patients suffering from genetic LPL deficiency, was a first attempt to treat the risk of pancreatitis (Fig. 1) [15]. The promise to lower plasma triglycerides with a single intramuscular application of adeno-associated virus serotype 1 harbouring the genetic code to locally produce LPL did however not become true in the current clinical practice. The drug has been registered for 2 years but is no longer available for treatment [40]. Though off the market, Glybera is one of the few drugs tested on postprandial chylomicron metabolism in addition to fasting triglycerides levels, with favourable results in patient suffering from LPL deficiency [41]. Prospective studies recognize postprandial triglyceride levels as risk factor in atherogenesis [42, 43]. Considering this, it will be interesting to evaluate whether the newly tested triglyceride-lowering drugs also affect postprandial hypertriglyceridemia. Up till now, however, published data mainly address fasting triglyceride levels and thus leave us speculating about postprandial effects.

### DGAT1 Inhibition

Diacylglycerol acyltransferase1, DGAT1, is an enzyme that catalyses the esterification of the third fatty acid to diacylglycerol to form a triglyceride molecule [44]. DGAT1 is ubiquitously expressed, though mostly in the duodenum and small intestine. Inhibition of DGAT1 was previously shown to have anti-obesity and anti-diabetic effects in 74 overweight or obese but otherwise healthy subjects [45]. A phase 2 trial with 8 patients with FCS showed triglycerides reductions up to 70% upon treatment with the DGAT1 inhibitor Pradigastat [46]. Subsequent phase 2 (2014) and phase 3 (2015) studies in patients with coronary artery disease and hypertriglyceridemia or FCS respectively were, however, prematurely terminated following interim analyses. Surprisingly, no information on the actual reasons for termination could be found in on the clinical trial pages (NCT01589237 and NCT01474434). In the public domain, there is no information or plan for using this compound to treat hypertriglyceridemia. Pradigastat may, however, still serve other purposes: in view of the notion that loss-of-function mutations in *DGAT1* have been associated with diarrhoea due to impaired intestinal fat absorption [44], a phase 3 trial is currently testing moderate impairment of intestinal fat absorption using Pradigastat in 180 patients with constipation (NCT04620161).

### FGF21

Fibroblast growth factor 21, FGF21, is a stress-inducible endocrine hormone primarily produced in hepatocytes that play important roles in regulating energy balance and glucose and lipid homeostasis. FGF21 analogues have been suggested to increase adiponectin levels, which stimulates fatty acid oxidation and thereby lowers liver and plasma triglyceride levels [47]. These FGF21 analogues have a longer half-life than the natural protein and have been tested in patients with hypertriglyceridemia but also in patients with NAFLD and non-alcoholic steatohepatitis (NASH) [47, 48].

In Phase 1a trial, a glycoPEGylated analogue of FGF21 (BIO89-100) has been shown to reduce plasma triglycerides by 33–51% [9–78 mg] in normolipidemic volunteers following a single subcutaneous injection [48]. In a subsequent Phase 1b/2a placebo-controlled, double-blind, multiple ascending dose study in patients with NASH, BIO89-100 has been shown to significantly reduce triglyceride levels by 18 to 28% [3–36 mg] and a further reduction of triglycerides by 33 to 49% [3–36 mg] in a subgroup with baseline TG ≥ 200 mg/dL [49••].

BIO89-100 is currently tested in 90 patients with severe hypertriglyceridemia (fasting triglycerides ≥ 500 mg/dL and ≤ 2000 mg/d L) in a phase 2 study. In the second quarter of 2021, BIO89-100 will be tested in patients with hepatic fibrosis stage 2 or 3 and NASH [50].

## Conclusions and Perspectives

Numerous studies have shown that recently registered drugs (Volanesorsen, Evinacumab) as well as experimental drugs have strong triglyceride-lowering potential. For proof of concept, the drugs have thus far been mostly used in patient groups with (very) high plasma triglycerides who are at increased risk of pancreatitis. Longer-term studies that have just been initiated will learn whether these drugs can address the unmet medical need to treat this life-threatening disorder.

Future trials may address the question whether any of these strategies can reduce the risk of ASCVD. A monoclonal antibody against ANGPTL3 (Evinacumab), is a good candidate in this respect as it is already registered for the treatment of hypercholesterolemia [32]. However, the other (experimental) drugs discussed in this review do not or are less potent to reduce cholesterol. It is in this regard tempting to indicate that the drugs that specifically lower plasma triglycerides and not cholesterol may ultimately answer the question whether plasma triglycerides cause atherosclerosis. Another point that merits attention, is that in the studies discussed here, only triglycerides levels in fasting plasma are reported with no mentioning of the possible effects on TRL, remnant TRL or remnant cholesterol [51]. With information in the public domain as only basis, it is therefore problematic to speculate on the possible impact of new triglyceride-lowering strategies on ASCVD. Naturally, such an effect will be welcome to treat ASCVD risk in patients with increased triglycerides in a context of obesity, T2DM and NAFLD.

In addition to potentially lower ASCVD risk, triglyceride-lowering drugs are also suggested to have beneficial effects on glucose metabolism and to reduce NAFLD. Since most studies have focused on the treatment of patients with severe hypertriglyceridemia, the data are too scarce to discuss this at length here. Early data have shown that use of AKCEA-ANGPTL3-LRx is not associated with changes in liver fat and HbA1c [37•]. On the other hand, the first studies with the FGF21 agonist BIO89-100 show that marked reduction of triglycerides were accompanied by reductions in liver fat, as well as improvements of glucose homeostasis [49••].

To date, it is tempting to speculate whether any of the treatments discussed here may ultimately become available for patients other than those suffering from orphan diseases such as FCS or HoFH. From the studies discussed in this review, BIO89-100, an FGF21 agonist, shows most promise in cardiometabolic patients. When considering the actual costs of any treatment per year per patient in the next few years, it is likely that macromolecule drugs (monoclonal antibodies) may not be able to compete with small molecule drugs in the long term (see Table 2). In the future, it will hopefully be possible for physicians and patients to discuss whether to choose subcutaneous injections or intravenous injections as a preferred choice to reduce plasma triglycerides to treat their medical problem [52].

**Table 2:** Overview of triglyceride-lowering drug specifications

Target	Drug	Route of administration	Drug use regimen / Duration of intervention	Notes	Pricing (May, 2021)	Reference
APOC3	Volanesorsen *ASO*	Subcutaneous injection	Weekly / maximum of 52 weeks tested	Combined with dietary interventions Side effects: injection site reactions, thrombocytopenia, immunogenicity	€903.819,80 patient/year (the Netherlands) [53]	Witztum et al. (2019) [20]
	AKCEA-APOC3-LRx *GalNac-ASO*	Subcutaneous injection	Weekly, biweekly or monthly / maximum of 52 weeks tested	Side effects: mild injection site reactions	n.a	Ionis pharmaceuticals (2020) [23•], Alexander et al. (2019) [22]
	ARO-APOC3 *siRNA*	Subcutaneous injection	Single injection / 16 weeks tested	Side effects: mild injection site reactions, moderate transient ALT elevation	n.a	Ballantyne (2020) [25], Arrowhead pharmaceuticals (2019) [27••]
	STT-5058 *mAb*	Intravenous injection	Biweekly, 3–6 doses in total / maximum of 14 weeks tested	Still recruiting	n.a	NCT04419688
ANGPTL3	Evinacumab *mAb*	Intravenous injection	Monthly / maximum of 24 weeks tested	Combined with existing lipid-lowering regimens* Side effects: nasopharyngitis, influenza-like illness, dizziness, rhinorrhoea, and nausea. May cause harm to fetus	$450,000 patient/year (USA) [54]	Raal et al. (2020) [34•]
	IONIS-ANGPTL3_LRx *GalNAc-ASO*	Subcutaneous injection	Monthly / maximum of 27 weeks tested	Side effects: mild injection site reactions, dizziness, headache	n.a	Gaudet et al. (2020) [37•], Graham et al. (2017) [36]
	ARO-ANG3 *siRNA*	Subcutaneous injection	Single injection / 16 weeks tested	Side effects: mild injection site reactions, mild transient ALT elevations	n.a	Watts (2020) [38], Arrowhead pharmaceuticals (2019) [27••]
DGAT1	Pradigastat *DGAT1 inhibitor*	Oral administration	Daily / 3 weeks tested	2 trials were terminated based on interim results (no details available) Side effects: mild, transient gastrointestinal adverse events	€396.396 patient/year (the Netherlands) [55]	Meyers et al. (2015) [46]
FGF21	BIO89-100 *FGF21 analog*	Subcutaneous injection	Biweekly / 12 weeks tested	Side effects: mild injection site reactions and headache	n.a	Frias et al. (2021) [49••]

Abbreviations: ALT, alanine aminotransferase; APOC3, apolipoprotein C3; ANGPTL3, angiopoietin-like protein 3; ASO, antisense oligonucleotide; DGAT1, diacylglycerol acyltransferase 1; FCS, familial chylomicronemia syndrome; FGF21, fibroblast growth-factor 21; GalNAc, N-acetylgalactosamine; mAb, monoclonal antibody; n.a, not available; siRNA, single-interfering RNA

^*^Evinacumab is used as treatment for HoFH in combination with the following existing lipid-lowering therapies: statins, fibrates, PCSK9 inhibitors, ezetimibe, lomitapide and apheresis

The studies discussed in this review are in part published in peer-reviewed journals, but we have also provided early information that has thus far only been shared at scientific meetings and through web pages of the respective pharmaceutical companies. We nevertheless want to highlight that several new strategies are within reach for patients with severe dyslipidaemia. The promise to decrease ASCVD risk in HoFH (Evinacumab) or decrease risk of pancreatitis in patients with FCS or severe hypertriglyceridemia (Volanesorsen or Evinacumab) is real and meets a distinct clinical need. With more drugs at hand, it will even be possible to truly tailor medical care to the individual patient. For example, a patient suffering from FCS should be genetically diagnosed prior to describing APOC3 or ANGPTL3 antagonists but at what costs? Another question that remains to be answered is whether any of the drugs described here will decrease the risk of atherosclerosis and proof that TRL is indeed atherogenic. To our knowledge, dedicated studies to answer this question have yet to be initiated.
